# Chemical Removal of Lead Corrosion Products

**DOI:** 10.3390/ma13245672

**Published:** 2020-12-12

**Authors:** Jan Švadlena, Tomáš Prošek, Kristýna Charlotte Strachotová, Milan Kouřil

**Affiliations:** 1 Department of Metallic Construction Materials, Technopark Kralupy of the University of Chemistry and Technology Prague, Nám. G. Karse 7, 278 01 Kralupy nad Vltavou, Czech Republic; tomas.prosek@vscht.cz; 2 Department of Metals and Corrosion Engineering, University of Chemistry and Technology Prague, Technická 5, 166 28 Prague, Czech Republic; strachotova.tyna@gmail.com (K.C.S.); kourilm@vscht.cz (M.K.)

**Keywords:** lead, restoration treatment, corrosion products, mass loss measurement

## Abstract

Restoration treatment, specimen preparation or mass loss measurements on coupons made of lead require a reliable process of dissolution of corrosion products. In this study, several types of model corrosion products with compositions representative of those found on real objects were prepared and characterized. Ten solutions were then thoroughly tested in interval cleaning experiments, regarding the efficiency of removal of the corrosion products, corrosivity towards bare lead, and remnants left on the surface. The solution recommended in the current version of the ISO 8470 standard was found to be improper for the cleaning of both historical artefacts and corrosion coupons due to its inability to remove sulfide corrosion products and the risk of surface contamination and staining. A solution of 20% hydrochloric acid is the best choice for the preparation of lead coupons before exposure or for evaluation of mass loss of exposed samples because its somewhat higher corrosivity towards metallic lead is tolerable for these applications. Rochelle salt solution was found to be optimal for the cleaning of historical artefacts free of sulfide corrosion products due to the lowest corrosivity. None of these alternative solutions leave remnants on the surface and they are efficient at laboratory temperature.

## 1. Introduction

In common atmospheric environments, lead is a very stable metal with low corrosion rates. Corrosion products in atmospheric conditions form thin and protective layers, strongly limiting corrosion of the underlying metal. Composition of the corrosion products depends on the exposure conditions and presence of pollutants. Typical lead corrosion products include various modifications of oxides and carbonates. Chlorides, sulfides, and organic compounds such as acetates and formates can form in presence of corresponding pollutants [1,2,3]. An overview of lead corrosion products summarized from references [4,5] with mineral names is given in Table 1.

Lead (II) oxide, PbO, is usually the first product of corrosion to appear in a clean atmosphere. In the presence of CO_2_ and humidity, a thin layer of acidic electrolyte forms on the surface and reacts with PbO to produce more thermodynamically stable lead carbonates, either cerussite, hydrocerussite or plumbonacrite [1,2,3,4,5,6,7,8,9,10,11]. If the atmosphere contains sulfur compounds, lead sulfide or lead sulfates may be present in the corrosion products [1,9].

Specific corrosion stimulants for lead are volatile organic compounds (VOCs), especially organic acid vapors. As shown in laboratory studies, organic acids vapors are highly corrosive to lead even at concentrations as low as 0.1 ppm [12,13]. Organic acids are able to disrupt protective corrosion films by condensation on corroded surfaces or in defects of the protective films and subsequently dissolve the original corrosion products, e.g., carbonates [1,13]. Newly formed corrosion products based on unstable organic acid salts lack protective properties of the former layers. Of organic acids that may be present in atmosphere, acetic acid has the most corrosive effect. The presence of volatile organic compounds and organic acids in higher concentrations is more common for indoor atmospheres. For example, concentrations of acetic acid in museum environments can be up to 20 times higher than in outdoor atmosphere [3]. Wood, adhesives, and plastics are well-described sources of the organic acids. In the environment of depositaries, specific materials such as old archival boxes, degraded documents, or wood and plywood furniture can emit significant amounts of acetaldehyde, acetic acid, formic acid and formaldehyde into an enclosed atmosphere with low air circulation [14].

Because of the protective nature of lead corrosion products, removal of these layers is usually done only when it is necessary for further restoration treatment or when they lose the protective properties, e.g., due to exposure to air polluted with acetic acid vapors [1]. Voluminous and porous corrosion products, mainly thick layers of cerussite, hydrocerussite or plumbonacrite, which lack protective properties, are usually removed as a part of conservation treatment to ensure safe conditions for long-term storage, due to aesthetical reasons, or when an application of a protective organic coating is considered [1,2,3]. However, it should be noted that in common atmospheric conditions, lead corrosion products work as a protective layer on historical artefacts. Therefore, it is not a common practice to remove them unless necessary.

Another reason for removing lead corrosion products is to assess mass losses and the corrosion rates of lead coupons after exposure. They can be used for the evaluation of corrosivity of indoor atmospheres as defined in the updated ISO 11844-1 [15] standard following the methodology described in ISO 11844-2 [16].

Three similar procedures for removal of lead corrosion products were given in the standard ISO 8407 version from 1991 [17]:

- Solution (1000 mL) of acetic acid (10 mL) in distilled water at boiling temperature.
- Solution (1000 mL) of ammonium acetate (50 g) in distilled water at 60–70 °C.
- Solution (1000 mL) of ammonium acetate (250 g) in distilled water at 60–70 °C.

Revised version of the standard published in 2009 replaced the three former solutions with a single one [18]. The same solution is proposed in the new ISO 11844-2 [16]:

- Saturated solution of ammonium acetate (1500 g in 1000 mL of distilled water) at laboratory temperature (20–25 °C).

Apart from the listed solutions, several other methods of chemical cleaning are mentioned in literature, including hydrochloric acid in concentrations up to 20% [9], chelating agent ethylenediaminetetraacetic acid (EDTA) and synthetic ion-exchange resin [1].

Key factors for selecting the right cleaning procedure are the ability to remove all types of possible corrosion products and low corrosivity towards bare metal. For the treatment of historical objects and preparation of corrosion coupons, the surface is additionally required to stay free from potentially corrosive remnants of the cleaning agent. When selecting a cleaning solution for the preparation of samples before exposure, the ability to remove all types of possible corrosion products and to leave the surface without any remnants of the reagents are the most important factors, while increased corrosivity towards bare metal is not an issue. Therefore, different solutions might be suitable for specific applications.

As already mentioned, acetic acid and acetates are highly corrosive for lead, and, especially in combination with required elevated temperatures, render the use of the standardized cleaning procedures problematic. The aim of this work was to compare the standard solutions for the removal of lead corrosion products with alternatives in view of their corrosion products removal efficiency, corrosivity towards metallic lead, danger of surface contaminations with their remnants, and operational complexity.

## 2. Materials and Methods

Lead sheet (99.99%) cut into 10 mm × 50 mm coupons of 0.5 mm thickness served as a substrate for the preparation of corrosion products in order to evaluate the ability of cleaning solutions to remove corrosion layers of different compositions. The coupons were abraded, pickled in 20% hydrochloric acid, thoroughly washed with deionized water, and degreased in acetone. Just before the exposure, the surface of the coupons was once again abraded, this time with an abrasive wadding (3M Scotch-Brite CF-MF), rinsed with acetone, air-dried and weighed. To form desired types of model corrosion products, the coupons were exposed under specific conditions listed below:

- Oxide corrosion products: coupons were exposed in a climatic chamber at 75 °C with air saturated with water (condensation conditions at 100% relative humidity, RH) for 14 days.
- Sulfide corrosion products: coupons were exposed in an atmosphere with about 100 ppm H_2_S at high relative humidity of 90–95% at a laboratory temperature of about 22 °C for 14 days.
- Carbonate corrosion products: coupons were first immersed into 65 vol.% HNO_3_ for 20 min before being immersed without any rinsing into (NH_4_)_2_CO_3_ solution (5 g of (NH_4_)_2_CO_3_ in 100 mL of deionized water) at a laboratory temperature of about 22 °C for 24 h.
- Acetate corrosion products: coupons were exposed in an atmosphere above 20% solution of acetic acid at a laboratory temperature of about 22 °C for 7 days.
- Formate corrosion products: coupons were exposed in an atmosphere above 20% solution of formic acid at a laboratory temperature of about 22 °C for 7 days.

Composition of corrosion products was verified with infrared spectroscopy (Nicolet iS50, Thermo Fisher Scientific) using the Fourier-transform infrared/Attenuated total reflectance (FTIR/ATR) method, Raman spectroscopy (Nicolet iS50, Thermo Fisher Scientific) using 1064 nm wavelength laser, and X-ray diffraction (XRD) with the X’Pert PRO diffractometer (PANalytical, Almelo, The Netherlands). The aim of the analyses was to confirm that the required type of model corrosion products dominated on the surface. Minor constituents were disregarded.

Ten cleaning solutions were chosen for removal of the model corrosion products, and are shown in Table 2. Three solutions were taken from the withdrawn version of the ISO 8407 standard [17], one solution from the 2009 revised standard version [18], three solutions contained different concentrations of hydrochloric acid, and two solutions contained different concentrations of EDTA disodium salt dihydrate, C_10_H_14_N_2_Na_2_O_8_·2H_2_O. A solution of so-called Rochelle salt, often used for treatment of bronze objects [19], was added as a possibly suitable cleaning agent because of promising preliminary results. It was prepared by dissolving 15 g potassium sodium tartrate, KNaC_4_H_4_O_6_·4H_2_O, and 5 g sodium hydroxide, NaOH, in 100 mL deionized water.

Removal of corrosion products and comparison of the efficiency of the tested solutions was based on the interval cleaning procedure described in the ISO 8407 standard [18], which allows for determination of the rate of corrosion products dissolution. A specimen was immersed into a given solution under ultrasonic stirring for a period of 30 s to 2 min at the beginning of the cleaning process, and up to 5 min in the end. The intervals were set for each group of pre-corroded samples based on the amount of corrosion products and pre-tests. After each partial cleaning step, the specimen was rinsed with acetone to remove remnants of the cleaning solution, air-dried to prevent any subsequent oxidation, and weighed. The procedure was repeated until reaching 10 min of the cleaning time in total. The same method was applied to a series of uncorroded freshly abraded specimens, which served as blanks. Finally, the specimens were analyzed with XRD, Raman spectroscopy or FTIR to detect any residues of corrosion products or cleaning solutions.

To assess the corrosivity of cleaning solutions to bare lead, freshly prepared 10 mm × 50 mm specimens were exposed in the solutions for 200 min and mass loss was measured.

## 3. Results

### 3.1. Characterization of Corrosion Products

**Figure 1:**
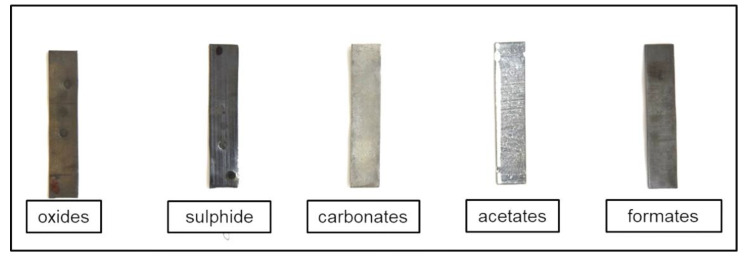
Appearance of lead coupons with prepared model corrosion products.

Quantities of the prepared corrosion products were assessed using gravimetry. Prepared layers of oxide corrosion products were thin according to the obtained interval cleaning curves. There was 2 to 5 g·m^–^² of oxide-based corrosion products on the surface. The exact composition of the oxide layer was not identified. The layer was darker compared to the bare lead surface and only slight tonal change towards orange or red typical for thicker lead oxide films was observed (Figure 1).

The thin sulfide corrosion products formed during the exposure in humid air with 100 ppm H_2_S blackened the surface, although their coverage was only 1 to 3 g·m^−^². The presence of the sulfide layer was confirmed indirectly because PbS is undetectable in typical FTIR instruments, the only infrared-active absorption peak being at 166 cm^−1^ [20]. XRD could also not detect it due to a lack of crystallinity or low thickness. The FTIR measurements served for confirmation that lead sulfate, PbSO_4_, was not present. Combining visual observation of the darkened surface and the selected exposure conditions, it was possible to reliably claim that the surface layer consisted mainly of lead sulfide.

White films of carbonate corrosion products, covering the lead surface with 5 to 10 g·m^−^², consisted mainly of cerrusite with 2–3 wt.% of hydrocerrusite, according to the XRD analysis (Table 3).

**Figure 2:**
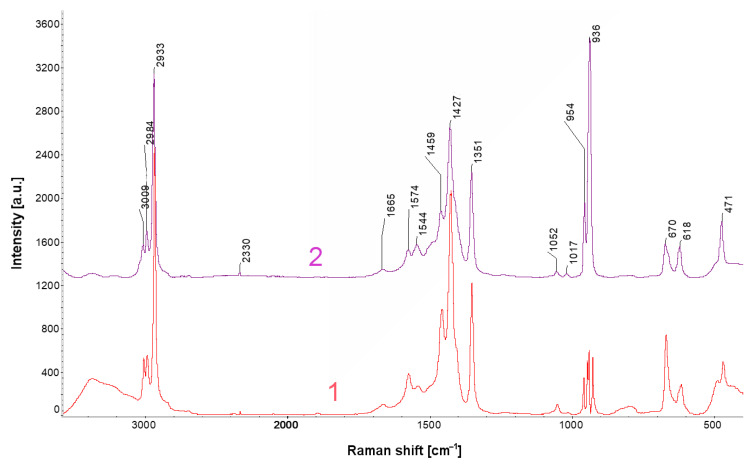
Comparison of Raman spectra of model lead acetate corrosion products (1) and lead acetate, Pb(CH_3_COO)_2_, standard (2).

The amounts of acetate and formate corrosion products varied significantly at 10–40 g·m^−^² and 1–20 g·m^−^², respectively. Acetate corrosion products caused surface whitening, while formate layers were darker gray. Raman spectra of the acetate corrosion products and Pb(CH_3_COO)_2_ standard in Figure 2 show a close similarity.

### 3.2. Removal of Corrosion Products

**Figure 3:**
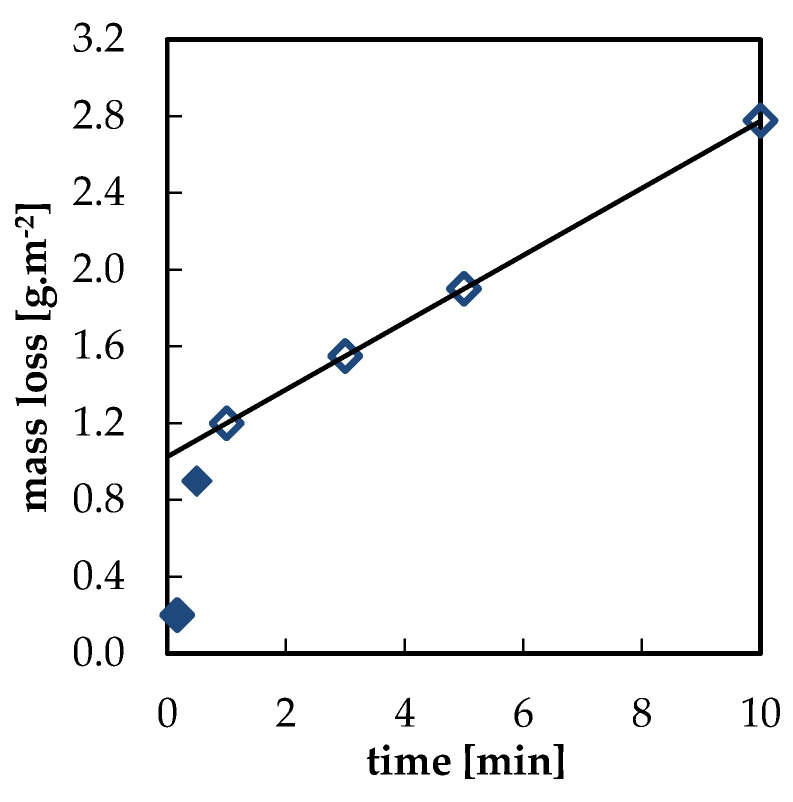
Interval cleaning of blank specimen in 1% HCl water solution; ⧫ dissolution of an oxide layer; ◊ dissolution of metallic lead.

**Figure 4:**
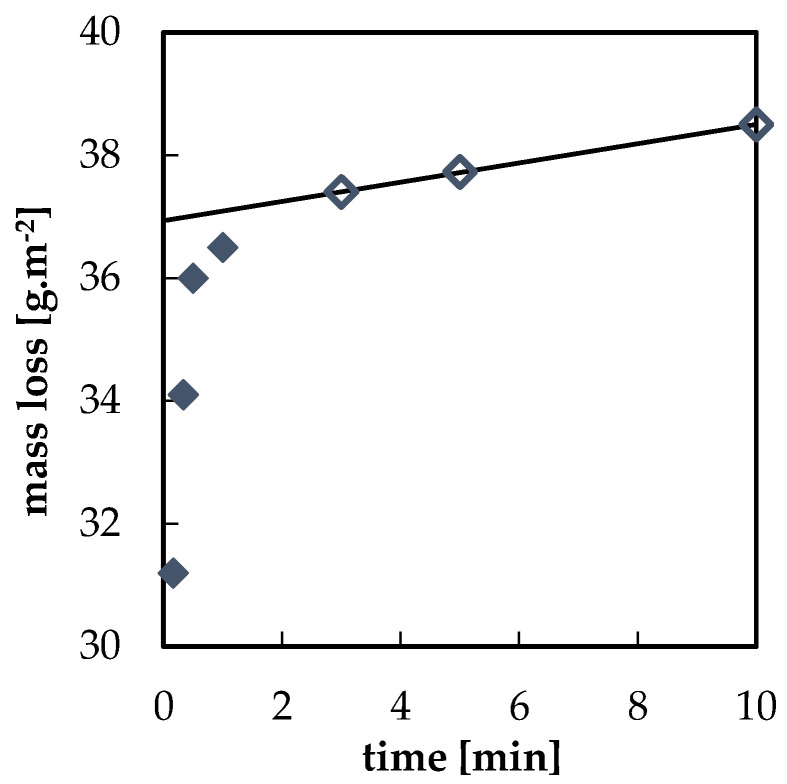
Interval cleaning of specimen with acetate corrosion products in 1% HCl water solution; ⧫ dissolution of corrosion products; ◊ dissolution of metallic lead.

The prepared model corrosion products were removed from the samples using interval cleaning. Examples of interval cleaning data in 1% HCl for bare lead and lead with acetate corrosion products are shown in Figure 3 and Figure 4, respectively. Dependences of mass loss on time in tested solutions showed two clearly distinguishable regions. The first data points, which are represented by full symbols, correspond to the rapid removal of corrosion products. Data points obtained at the end of the process are linked to the slower dissolution of non-corroded lead (empty symbols). Following the ISO 8407 standard, it is possible to assess the amount of corrosion products removed from the surface before metal dissolution as the intercept of the y-axis and the extrapolated line segment interlaying the latter points. The rapid drop in mass during the first minute of the pickling process of the blank freshly abraded specimen displayed in Figure 3 can be seen as proof of the presence of a thin oxide film formed by instantaneous oxidation of the abraded lead surface even in laboratory conditions. Indeed, a thin oxide layer is always present on the lead surface [7,21].

**Figure 5:**
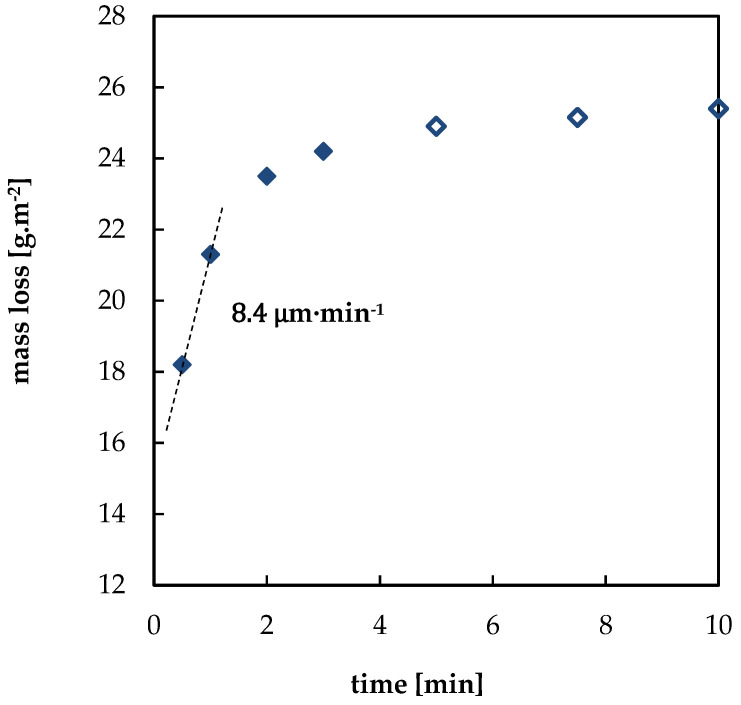
Determination of the rate of corrosion products dissolution (dashed line) for a specimen with acetate corrosion products in saturated ammonium acetate solution; ⧫ dissolution of corrosion products; ◊ dissolution of metallic lead.

However, in this study, the main parameter obtained from the interval cleaning curves was the rate of corrosion product dissolution. This was determined from the slope of the line segment fitted to the initial mass loss points, as shown in Figure 5. All data on corrosion products dissolution rates are summarized in Table 4. For most combinations of the type of corrosion products and cleaning agent, the rate of corrosion product removal was sufficiently high, and the specimen surface was completely clean after 3 to 10 min. Oxide and sulfide corrosion products showed low dissolution rates, suggesting that these types of corrosion products may be more difficult to remove. The latter type of corrosion products, especially, dissolved slowly, and the dissolution rate was too low to be measurable in several solutions. In contrary, acetates dissolved readily in all solutions.

Table 5 summarizes the effectiveness of each solution for removing specific corrosion products. The data are based on FTIR, Raman and XRD analyses of lead specimens after the cleaning procedure and the data on the rate of corrosion product removal given in Table 4. In some cases, corrosion products were removed only partially within the 10 min cleaning period. For specimens with oxide corrosion products cleaned in 1% and 10% hydrochloric acid solution and 5% ammonium acetate solution, it was verified that a prolonged exposure of 30 min led to complete removal of the corrosion products. Although it is probable that prolonged cleaning would also be efficient in most other cases, it is impractical and risky because it can inflict pronounced corrosion of the base metal.

As shown in Table 4 and Table 5, oxide and sulfide corrosion products were difficult to remove in many cases. Only a part of the thin layer of lead oxides was possible to remove with diluted hydrochloric acid (1% and 10% solutions), 5% ammonium acetate and EDTA solutions during 10 min of cleaning. Prolonged time in the solutions then caused dissolution of the underlying lead rather than lead oxides. Layers of lead oxides were completely removed in more concentrated ammonium acetate solutions, boiling acetic acid solution, 20% hydrochloric acid and solution of Rochelle salt.

Although lead (II) sulfide is not commonly found on corroded lead objects, its presence even as a thin layer has a distinct influence on the lead corrosion behavior [2,7]. Only hydrochloric acid was able to remove it completely.

Other corrosion products were successfully removed from the specimens within 10 min of cleaning in most cases. The most efficient removal of all types of corrosion products was recorded for a 20% solution of hydrochloric acid.

### 3.3. Residues of Cleaning Solutions on Lead Surface

**Figure 6:**
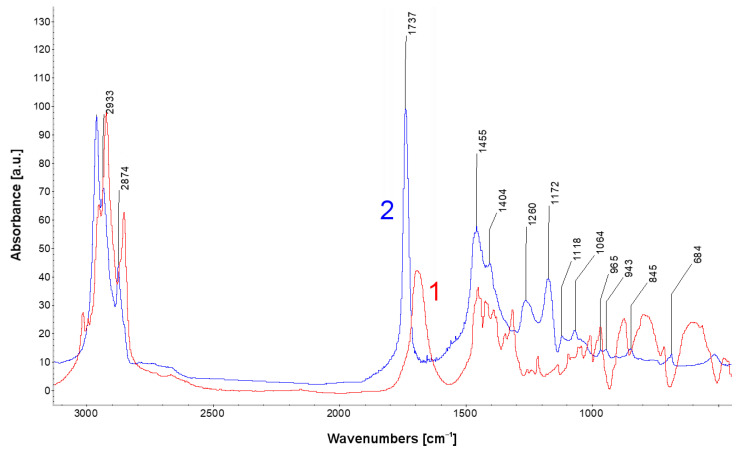
FTIR/ATR spectrum of lead specimen after cleaning with EDTA (1) compared with spectrum of EDTA acid (2).

After the treatment with EDTA solutions, residues of the chelating agent were detected on the surface with FTIR/ATR analysis. FTIR spectra of a specimen with EDTA remnants and EDTA standard are shown in Figure 6. Absorption peaks in the area from 2800 cm^−1^ to 3000 cm^−1^ confirm the presence of –CH_2_– groups of an organic compound. Characteristic R–COO– acid group absorption around 1700 cm^−1^ is evident in both spectra. Certain shifts in the absorption peak positions in the sample spectrum were possibly caused by partial resonance of –CO groups. The fingerprint region under 1450 cm^−1^ is usually difficult to assign for large organic molecules and many drifts in the peak positions will occur with respect to how the molecules are bound to the metallic surface.

Several methods of sample rinsing after the treatment were tested, including rinsing with acetone, deionized water and ethanol and deionized water and acetone in an ultrasonic bath. In all cases, traces of EDTA were detected on the surface. This is highly undesirable and can cause oxidation of the metal surface after the treatment and affect further corrosion and appearance of lead.

After cleaning in hydrochloric acid solutions, thorough rinsing with acetone followed by air-drying was sufficient for removal of all residues of cleaning solution and minimalizing the risk of further oxidation. Several additional steps of rinsing with deionized water were required to remove all traces of acetic acid and ammonium acetate solutions completely. However, prolonged rinsing with deionized water caused the immediate oxidation of lead, leaving the surface stained and possibly altering the results of mass loss determination. Another reason for rapid surface oxidation during cleaning was the elevated temperature.

### 3.4. Corrosiveness of Cleaning Solutions to Bare Lead

A solution for chemical cleaning should ideally remove all corrosion products without any dissolution of the underlying metal. In practice, some limited dissolution of metal always occurs. However, the rate of metal dissolution as well as the ratio of metal and corrosion products dissolution rates serve as important criteria for the selection of an optimal procedure for chemical cleaning. Corrosivity of cleaning solutions towards bare metal was assessed gravimetrically after the exposure of freshly prepared lead samples to the tested solutions for 200 min. Preliminary tests proved that this exposure period was long enough for the stabilization of a corrosion rate. The results are compared in Table 6 using corrosion rates (*r_corr_*) calculated from mass losses using equation

$$r_{corr}=\frac{\Delta m\cdot A}{\rho \cdot t},$$

where Δ*m* is mass loss, *A* represents the surface area of the sample, *ρ* is the density of lead, and *t* is time of the exposure. The presented values of corrosion rates are averages of three experiments.

Susceptibility of lead to increased dissolution in the presence of hot acetic acid or acetates manifested itself, as expected, in elevated corrosion rates in the respective solutions. The corrosion rate of saturated solution of ammonium acetate was significantly lower, mainly because of the lower working temperature. Rochelle salt, saturated ammonium acetate and EDTA solutions showed the lowest corrosion rates. For hydrochloric acid, corrosion rates in 1% and 10% solutions were similar, whereas it was somewhat elevated in the 20% solution.

## 4. Discussion

Several solutions can be rejected as unsuitable. EDTA failed to remove sulfide corrosion products and left undesirable residues on the sample surface. The 1% acetic acid solution was operated at an elevated temperature, which poses risks of subsequent oxidation during sample rinsing in deionized water. Rinsing in acetone was unable to remove residues of the cleaning solution. Complete removal of sulfide corrosion products was not achieved. Diluted 5% and 25% ammonium acetate solutions also necessitate elevated working temperatures and sample rinsing in deionized water, and they were unable to remove sulfide corrosion products.

A suitable cleaning solution should show a sufficient difference between the rates of corrosion products and bare metal dissolution. In Table 7, ratios of these rates are presented for the solutions that were not excluded in the previous step. A higher number means a larger difference between the rates, i.e., a more desirable result. High values for acetate corrosion products were expected because they are readily soluble. The rate of corrosion product dissolution relatively to the rate of metal dissolution was high in 20% hydrochloric acid. A saturated solution of ammonium acetate and solution of Rochelle salt have similar ratios for all types of the corrosion products.

All results are combined in Table 8 with respect to the applicability of the solutions for the removal of corrosion products from lead objects and coupons. The Remaining Corrosion Products column states the type of corrosion product that remained on the surface after the cleaning. Risk of Surface Contamination with residues of the cleaning solution is an important factor to take into account when choosing the proper solution to prepare samples or to achieve a clean and stable lead surface of, for example, historical artefacts. It is not that significant for the determination of mass loss and corrosion rate on lead coupons after exposure in atmosphere of interest. Risk of Subsequent Oxidation focuses on the possible oxidation of cleaned lead surfaces during rinsing and drying. This may occur mainly because of elevated working temperature for some of the cleaning procedures, or when rinsing in deionized water is required to remove traces of cleaning solutions. Operational Complexity deals with requirements for the cleaning procedure, such as the necessity for solution heating or complex sample rinsing.

Somewhat higher corrosion rates of non-corroded lead in 20% hydrochloric acid solution renders the solution dangerous for the safe removal of corrosion products for a purpose of conservation treatment. However, its reliability in the removal of all types of lead corrosion products, no risk of surface contamination, and easiness of operation make this solution optimal for sample preparation and evaluation of mass loss of coupons.

A saturated solution of ammonium acetate, the only cleaning solution for lead in current version of the standard ISO 8407, was not efficient enough in removing sulfide corrosion products. It could be disregarded given that sulfide corrosion products are not common on historical lead artefacts. However, ammonium acetate remnants were impossible to remove from the surface by rinsing with acetone and several additional steps of rinsing with deionized water had to be applied, increasing the operational complexity and risk of surface oxidation and staining.

Rochelle salt, achieving the lowest corrosion rate towards bare lead, low operational complexity and low risk of surface contamination and subsequent oxidation, is the best substitute to the saturated ammonium acetate solution for the conservation treatment of historical objects, especially when further reduction in the risk of lead substrate dissolution is required. It was also more efficient in the removal of lead oxide than hydrochloric acid.

In the presence of lead sulfide, diluted hydrochloric acid can be considered as an alternative to Rochelle salt for the conservation treatment of lead historical objects. The only drawback of these solutions is a somewhat lower efficiency against thicker layers of lead oxides. Gravimetric measurements showed that corrosivity of 1% and 10% solutions of hydrochloric acid towards bare lead was sufficiently low. Because the 1% solution was prone to rapid depletion when used for removal of more voluminous corrosion products and slightly more corrosive, the 10% solution is seen as a better option for this purpose.

## 5. Conclusions

Thorough comparison of ten solutions for removal of lead corrosion products in view of their efficiency, corrosion risk and operational complexity allowed for drawing of the following conclusions:

- Saturated ammonium acetate, the only cleaning solution for lead in the current version of the standard ISO 8407, is unable to remove sulfide corrosion products and leaves remnants on the surface after rinsing with acetone or induces risks of subsequent surface oxidation and staining when rinsed with demineralized water. It cannot be recommended for the cleaning of either historical artefacts or corrosion coupons.
- A solution of 20% hydrochloric acid is the best choice for the preparation of lead coupons before exposure or for the evaluation of mass loss of exposed samples. It is able to remove all types of corrosion products, leaves no remnants, and it is easy to use. However, elevated corrosivity towards metallic lead makes it less suitable for the cleaning of historical objects.
- Rochelle salt solution was found to be optimal for the cleaning of historical artefacts due to its lowest corrosivity towards metallic lead. It cannot dissolve lead sulfide, but it is mostly acceptable given that the sulfide corrosion products are not common on historical lead artefacts.
- In the presence of lead sulfide, 10% hydrochloric acid can be considered as an alternative to Rochelle salt despite a medium corrosivity towards metallic lead.
- Boiling acetic acid, EDTA solutions, and 5% and 25% ammonium acetate solutions were determined as unsuitable due to serious drawbacks including aggressiveness to the lead substrate, inability to remove certain types of corrosion products, and the presence of cleaning solution residues on the surface.

## Figures and Tables

**Table 1:** Lead corrosion products [4,5].

Compound	Mineral	Formula
**Lead (II) oxide**	massicote	α-PbO
	litharge	β-PbO
**Lead (IV) oxide**	plattnerite	PbO_2_
**Lead carbonates**	cerussite hydrocerussite plumbonacrite	PbCO_3_ Pb_3_(CO_3_)_2_(OH)_2_ Pb_10_(CO_3_)_6_O(OH)_6_
**Lead (II) chloride**	cotunnite	PbCl_2_
**Lead (II) sulfide**	galena	PbS
**Lead (II) sulfate**	anglesite	PbSO_4_
**Lead acetates**	–	Pb(CH_3_COO)_2_ Pb(CH_3_COO)_2_·3H_2_O Pb(CH_3_COO)_2_·Pb(OH)_2_·H_2_O Pb(CH_3_COO)_2_·2PbO·H_2_O
**Lead formates**	–	Pb(CHOO)_2_ Pb(CHOO)(OH) Pb(C_4_H_4_O_5_)·H_2_O

**Table 2:** Tested cleaning solutions for removal of model corrosion products.

Solution (Aqueous)	Concentration	Conditions
Acetic acid	1%	Boiling
Ammonium acetate	5%	60–70 °C
	25%	60–70 °C
	saturated	Laboratory temperature, ≈22 °C
Hydrochloric acid	1%	Laboratory temperature, ≈22 °C
	10%	Laboratory temperature, ≈22 °C
	20%	Laboratory temperature, ≈22 °C
EDTA	1%	Laboratory temperature, ≈22 °C
	10%	Laboratory temperature, ≈22 °C
Rochelle salt (15 g KNaC_4_H_4_O_6_·4H_2_O + 5 g NaOH in 100 mL)		Laboratory temperature, ≈22 °C

**Table 3:** XRD analysis of the model carbonate corrosion products on lead coupons.

Compound Name	Mineral Name	Chemical Formula	Estimated Quantity [wt.%]
Lead carbonate	Cerussite	PbCO_3_	71
Lead	Lead	Pb	27
Lead carbonate hydroxide	Hydrocerussite	Pb_3_(CO_3_)_2_(OH)_2_	2

**Table 4:** Dissolution rate of model corrosion products in tested solutions.

Solution	Concentration	Rate of Dissolution [µm·min^−1^]				
		Oxides	Sulfide	Carbonates	Acetates	Formates
Acetic acid	1%	0.2	0.2	2.1	1.1	1.3
Ammonium acetate	5%	0.5	Low	0.9	2.4	0.3
	25%	0.4	0.2	2.7	9.4	1.2
	saturated	0.3	Low	0.2	8.4	0.7
Hydrochloric acid	1%	0.2	0.2	1.4	26.1	0.5
	10%	0.1	0.1	0.9	29.6	0.3
	20%	0.4	0.5	2.1	4.2	2.3
EDTA	1%	0.3	Low	0.8	0.9	1.4
	10%	0.2	Low	0.7	1.0	1.3
Rochelle salt	–	0.3	Low	0.5	11.8	1.3

Low: Dissolution rate too low to be measurable.

**Table 5:** Effectiveness of cleaning solutions to remove specific corrosion products.

Solution	Concentration	Oxides	Sulfide	Carbonates	Acetates	Formates
Acetic acid	1%	✓	(✓)	✓	✓	✓
Ammonium acetate	5%	(✓)	X	✓	✓	✓
	25%	✓	(✓)	✓	✓	✓
	saturated	✓	X	✓	✓	✓
Hydrochloric acid	1%	(✓)	✓	✓	✓	✓
	10%	(✓)	✓	✓	✓	✓
	20%	✓	✓	✓	✓	✓
EDTA	1%	(✓)	X	(✓)	✓	✓
	10%	(✓)	X	(✓)	✓	✓
Rochelle salt	–	✓	X	✓	✓	✓

X: Corrosion products not removed during 10 min of cleaning; (✓): Corrosion products partially removed during 10 min of cleaning; ✓: Corrosion products removed completely.

**Table 6:** Corrosion rates of freshly abraded lead in the tested solutions.

Solution	Concentration	*r_corr_* [µm·day^−1^]	Standard Deviation [µm·day^−1^]
Acetic acid	1%	4.4	1.7
Ammonium acetate	5%	10.4	1.6
	25%	20.0	2.0
	saturated	0.8	0.1
Hydrochloric acid	1%	2.5	0.1
	10%	1.9	0.1
	20%	3.6	0.2
EDTA	1%	1.6	0.2
	10%	0.8	0.2
Rochelle salt	–	0.5	0.1

**Table 7:** Ratios of corrosion products and bare metal dissolution rates.

Solution	Concentration	Oxides	Sulfide	Carbonates	Acetates	Formates
Ammonium acetate	saturated	18	NA	8	142	21
Hydrochloric acid	1%	14	12	62	443	16
	10%	9	7	40	503	8
	20%	24	26	94	71	71
Rochelle salt		16	NA	20	196	39

NA: Rate of corrosion products removal was too low to be measurable, therefore the ratio could not be obtained.

**Table 8:** Effectiveness of tested cleaning solutions to remove corrosion products from lead.

Solution	Concentration	Remaining Corrosion Products	Corrosivity towards Metallic Lead	Risk of Surface Contamination	Risk of Subsequent Oxidation	Operational Complexity
Acetic acid	1%	sulfide	moderate	moderate	high	high
Ammonium acetate	5%	sulfide, oxides	high	moderate	high	high
	25%	sulfide	high	moderate	high	high
	saturated	sulfide	low	moderate	moderate	moderate
Hydrochloric acid	1%	oxides	low	low	low	low
	10%	oxides	low	low	low	low
	20%	–	moderate	low	low	low
EDTA	1%	sulfide, oxides, carbonates	low	high	low	low
	10%	sulfide, oxides, carbonates	low	high	low	low
Rochelle salt	–	sulfide	low	low	low	low

Note: Properties given in red are undesirable.
